# Oxidative Stress: A Putative Link Between Lower Urinary Tract Symptoms and Aging and Major Chronic Diseases

**DOI:** 10.3389/fmed.2022.812967

**Published:** 2022-03-10

**Authors:** Zhenqun Xu, Rania A. Elrashidy, Bo Li, Guiming Liu

**Affiliations:** ^1^Department of Surgery, MetroHealth Medical Center, Case Western Reserve University, Cleveland, OH, United States; ^2^Department of Urology, Shengjing Hospital, China Medical University, Shenyang, China; ^3^Department of Biochemistry, Faculty of Pharmacy, Zagazig University, Zagazig, Egypt

**Keywords:** lower urinary tract symptoms (LUTS), oxidative stress, aging, chronic diseases, bladder

## Abstract

Aging and major chronic diseases are risk factors for lower urinary tract symptoms (LUTS). On the other hand, oxidative stress (OS) is one of the fundamental mechanisms of aging and the development of chronic diseases. Therefore, OS might be a candidate mechanism linking these two clinical entities. This article aims to summarize the studies on the prevalence of LUTS, the role of OS in aging and chronic diseases, and the potential mechanisms supporting the putative link. A comprehensive literature search was performed to identify recent reports investigating LUTS and OS in major chronic diseases. In addition, studies on the impact of OS on the lower urinary tract, including bladder, urethra, and prostate, were collected and summarized. Many studies showed LUTS are prevalent in aging and major chronic diseases, including obesity, metabolic syndrome, diabetes, cardiovascular disease, hypertension, obstructive sleep apnea, autoimmune diseases, Alzheimer’s disease, and Parkinson’s disease. At the same time, OS is a key component in the pathogenesis of those chronic diseases and conditions. Recent studies also provided evidence that exacerbated OS can cause functional and/or structural changes in the bladder, urethra, and prostate, leading to LUTS. The reviewed data support the concept that OS is involved in multiple risk factors-associated LUTS, although further studies are needed to confirm the causative relationship. The specific ROS/RNS and corresponding reactions/pathways involved in chronic diseases and associated LUTS should be identified in the future and could serve as therapeutic targets.

## Introduction

Lower urinary tract symptoms (LUTS) are highly prevalent worldwide, affecting up to 70% of adult men and women, depending on the study population, age, and methodology (1–4). LUTS comprise (i) bladder storage symptoms including frequency, urgency, urge incontinence, and nocturia, (ii) voiding symptoms such as hesitancy, low and/or intermittent stream, straining, prolonged micturition, and (iii) postmicturition symptoms such as the feeling of incomplete emptying and postmicturition dribble (4, 5). These symptoms significantly affect the quality of life and impose a high social and economic burden. LUTS can be caused by a local disease or injury in the lower urinary tract, such as urinary tract infection, prostatitis, urethral stricture, and bladder stones. In addition, numerous clinical and epidemiological studies have demonstrated that LUTS are common in aging and aging-related major chronic diseases and conditions (4, 6–9).

There is a variation in the definition of chronic diseases regarding the included diseases and their duration (10). Generally, chronic diseases refer to a physical or mental health disorder that exists for 3 months (or 1 year by some definitions) or more, causes functional restrictions, or requires continuous monitoring or therapy (10). Chronic diseases are common in the United States. It is estimated that 133 million Americans (or 45% of the population) suffer from at least one chronic disease, and this number is continuously increasing (11). LUTS were found to be highly prevalent in the aging population (12–16), and patients with chronic diseases and conditions such as obesity (17–21), metabolic syndrome (MetS) (22–26), diabetes (27–31), cardiovascular disease (32–36), hypertension (37–39), obstructive sleep apnea (OSA) (40–46), autoimmune diseases (47–50), Alzheimer’s disease (51–53), and Parkinson’s disease (54–57). These diseases seem to adversely affect one or more organs in the lower urinary tract (bladder and urethra) and prostate, leading to LUTS. However, the mechanistic links between LUTS and the aforementioned chronic diseases and conditions are unclear. Enhanced oxidative stress (OS) is a possible candidate mechanism since OS has been shown to be potentially implicated in the pathogenesis of all the above diseases. Therefore, OS may represent a unifying common pathogenic pathway between LUTS and chronic diseases.

Oxidative stress is an overload of oxidants and free radicals, mainly reactive oxygen species (ROS) such as superoxide radical anion (O_2_^–^), hydrogen peroxide (H_2_O_2_), hydroxyl radical (HO^–^), and reactive nitrogen species (RNS) including nitric oxide (NO) and peroxynitrite (ONOO^–^), due to increased production and/or compromised antioxidant defenses. The endogenous ROS are mainly produced in mitochondria, endoplasmic reticulum, peroxisomes, and phagocytic cells through enzymatic or non-enzymatic reactions (58). ROS/RNS can be removed by free radical scavengers in physiological conditions. The antioxidant enzymes include superoxide dismutase (SOD), glutathione peroxidase (GPx), glutaredoxins, thioredoxins, and catalase. The non-enzymatic antioxidants include glutathione (GSH), thioredoxin, lipoic acid, melatonin, carotenoids, uric acid, bilirubin, polyamines, and Vitamin E and C, etc.

Oxidative stress has long been believed to cause lipid, protein, and DNA damage affecting cellular functions, ultimately contributing to multiple pathological conditions. The level of OS can be measured indirectly by detecting the oxidative or nitrosative products of lipids (malondialdehyde, 4-hydroxy-2-non-enal), proteins (protein carbonyls, 3-nitrotyrosine), or nucleic acids (8-Hydroxy-deoxyguanosine). On the other hand, recent evidence has shown that low or moderate levels of ROS/RNS can serve as second messengers in intracellular signaling cascades involved in various cellular pathways (59).

This review described the prevalence of LUTS in aging and major chronic diseases (Table 1), the role of OS in the pathogenesis of these diseases and conditions, and the impact of OS on the lower urinary tract. We explored that OS may be a mechanistic link between LUTS and chronic diseases and hopefully aid internal medicine specialists and urologists to realize the potential connections between two clinical entities when they manage such patients.

**TABLE 1:** Summary of the findings on lower urinary tract symptoms (LUTS) in aging and chronic diseases.

Chronic condition	Study	Population	LUTS findings
Aging	Kupelian et al. (67)	5,506 aged 30–79 (2,301 men, 3,205 women)	18.7% overall, increased with age (10.5% at age 30–39 years vs. 25.5% at age 70–79 years). There is no gender difference
	Taylor et al. (63)	5,284 men, ≥65 year	LUTS were mild in 51.6%, moderate in 39.6%, and severe in 6.6%. The prevalence and severity increased with age
	Parsons et al. (62)	291 men (48.3–97.1 year)	56% in men < 80 year, 70% in men ≥ 80 year, and 90% in men ≥ 90 year
	Kim et al. (64)	1,842 men, ≥40 year	83.4% overall, 78.3% in men 40–49 year, and 89.6% in men aged ≥60 year
Obesity	Vaughan et al. (74)	3,727 aged 18–79 (53.7% women)	Obesity was associated with urinary frequency in men, stress urinary incontinence and urgency incontinence in women, nocturia in both men and women
	Penson et al. (75)	7,318 men aged 40–79 year	The risk for moderate to severe LUTS increased 38% in patients with a BMI ≥ 35 kg/m^2^
	Oliver et al. (76)	358 patients aged 6–17 years	Children with obesity had a higher mean score for LUTS (*p* = 0.009)
Metabolic syndrome (MetS)	Kupelian et al. (79)	1,899 men (30–79 year)	Men with mild to severe LUTS had higher odds of MetS (multivariate OR 1.68, 95% CI 1.21–2.35)
	Zamuner et al. (81)	490 men (36–84 year)	Men with MetS had an increased risk for LUTS. The odds ratio of MS having moderate or severe LUTS was 2.1
Diabetes	Van Den Eeden et al. (87)	184,646 men (18–79 year)	Type 2 diabetes is associated with a 1.32-fold increased risk of LUTS in men
	Bang et al. (88)	278 men (65.33 ± 9.05 year)	The International Prostate Symptom Score (IPSS) was higher in the type 2 DM group than in the control group (17.80 ± 7.60 vs. 15.88 ± 7.05; *p* = 0.031)
Cardiovascular diseases (CVDs)	Bouwman et al. (94)	6614 men, ≥50 year	41.1% in men with LUTS vs 19.5% in men without LUTS reported CVDs (*p* < 0.01)
	Tibaek et al. (97)	407 (45% women), ≥40 year	The overall prevalence was 94%. The most frequent symptom was nocturia (76%), followed by urgency (70%) and daytime frequency (59%)
Hypertension	Hwang et al. (37)	295 men (69.5 ± 7.0 year)	Men with hypertension had a higher IPSS-total (22.9 ± 7.8 vs. 21.2 ± 7.3, *p* = 0.01) and obstructive symptom score (13.3 ± 5.2 vs. 11.9 ± 4.7, *p* = 0.01) compared to control
	Chong et al. (103)	644 men, 40–87 year	Hypertension was significantly associated with moderate-to-severe LUTS (adjusted prevalence rate ratio = 1.626, 95% CI 1.029–2.570)
Obstructive sleep apnea (OSA)	Moriyama et al. (41)	73 men, 20–83 year	30 patients (41.1%) had nocturia. 22 patients were more than 50 years of age (50%), and 8 patients were 50 years old or less (27.6%)
	Chung et al. (43)	6180 men, ≥18 year	Men with OSA had higher prevalences of prostate hypertrophy (15.13 vs. 7.28%, *p* < 0.001), chronic prostatitis (4.37 vs. 2.16%, *p* < 0.001), urinary incontinence (3.32 vs. 0.87%, *p* < 0.001), and nocturia (2.02 vs. 0.61%, *p* < 0.001) compared to controls
Autoimmune diseases	Haarala et al. (47)	242 (36 SS, 85 SLE, and 121 control), 18–81 year	The prevalences of mild LUTS were 61%, 62% and 27%, and severe LUTS were 14%, 9% and 7% in the SS, SLE, and control group, respectively
	Zecca et al. (48)	403 (114 men, 289 women, 44.3 ± 11.6 year)	35% of MS patients had urine incontinence
Alzheimer’s disease (AD)	Lee et al. (110)	3,732 (933 AD, 2,799 control), ≥50 year	The risk of UI is higher in AD cohort (hazard ratio: 1.54, 95% confidence interval: 1.13–2.09) than in the control group
	Na et al. (52)	464 (339 women, 125 men), 78.43 ± 6.84 year	The prevalence of UI in AD patients was 24.8%. Urgency incontinence (44.3%) and functional incontinence (25.3%) were two most common types of UI
Parkinson’s disease (PD)	Campos-Sousa et al. (112)	135 (61 PD, 74 control, 36–83 year	LUTS prevalence was 39.3% in PD group vs. 10.8% in the control cohort. The most common symptom was nocturia, followed by frequency and urinary incontinence
	Hobson et al. (113)	215 (123 PD, 92 control, 56–92 year	The prevalence of urinary symptoms was 51% in PD patients vs. 30.6% in the control group

## Lower Urinary Tract Symptoms are Prevalent in Aging and Major Chronic Diseases

### Lower Urinary Tract Symptoms in Aging

According to the U.S. Census Bureau Data, 52 million (or 16% of the population) people are 65 or older in 2018; the number will reach 95 million by 2060 (60). Aging is the most significant risk factor for a wide range of chronic diseases. In the National Health Interview Survey performed by the Centers for Disease Control and Prevention (CDC) in 2008, 85.6% of the elderly population suffer from at least one chronic disease, and 56.0% experience at least two.

Urologic issues are ubiquitous in the elderly population and account for a greater number of clinic visits. It is estimated that about 50% of men at the age of 60 have histological benign prostatic hyperplasia (BPH)/LUTS, approximately 70–80% of men aged 60–89 years are affected, and by 90, the prevalence approaches 90% (61–63). Kim et al. (64) demonstrated that LUTS were typically seen in about 83.4% among 1,842 Korean men aged ≥40 years, and the prevalence and severity increased with age. In such a study, storage LUTS (70.1%) were more predominant than voiding LUTS (60.4%), while postmicturition LUTS occurred in 38.3% of the participants (64). Similar findings were obtained from another clinical study performed on 8,627 men aged 48–79 years old (65). Mild, moderate, and severe LUTS were reported in 75.3, 22.0, and 2.7% of patients, respectively, and the prevalence increased with age. Furthermore, age has been shown to be associated with the progression of LUTS in a population-based cohort of 5,502 participants ranging from 30 to 79 years old (66). A salient observation reported by Pöyhönen et al. (14) was that the most common LUTS among a population of men aged 30–80 years differed with increasing age, since men aged 30–40 years mainly complained of the dribble. While urgency and nocturia represented the most bothersome symptoms in men aged 70–80 years (14). In the Boston Area Community Health (BACH) Survey including both men and women, the prevalence of LUTS increased from 10.5% at age 30–39 years to 25.5% at age 70–79 years (67).

Although LUTS appears to be more prevalent in the elderly than in younger persons, they should not be considered a normal aspect of aging. Increased public awareness and appropriate therapeutic strategies should be adopted to manage these symptoms effectively.

### Lower Urinary Tract Symptoms in Obesity

Based on recent data released from the CDC: the prevalence of obesity increased from 30.5 to 42.4% from 1999 to 2000 through 2017–2018, and the prevalence of severe obesity increased from 4.7 to 9.2%. Increasing evidence suggests a link between obesity and LUTS. Obese patients are more likely to have LUTS, including voiding and storage symptoms as well as prostate enlargement in men than control subjects (20, 68). Increased waist circumference, as a manifestation of central obesity, is considered a predictive surrogate of the severity of LUTS in obese patients (69, 70) and animal models of obesity (71). Furthermore, central obesity has been recognized as a risk factor for storage problems and urinary incontinence after prostatectomy in patients with BPH (72). In contrast, surgical or behavioral weight loss results in modest improvements in urinary incontinence in obese women (73).

Vaughan et al. assessed the urinary storage symptoms in patients aged 18–79 years (74). They demonstrated that obesity was associated with nocturia in both men and women. Obesity was also associated with urinary frequency in men and stress urinary incontinence and urgency incontinence in women. Another study (75), including 7,318 men aged 40–79, found the risk for moderate to severe LUTS increased 38% in patients with a body mass index (BMI) of at least 35 kg/m^2^. Oliver et al. (76) prospectively evaluated 358 patients aged 6–17 years with LUTS using a 21-item questionnaire. They found that children with obesity had a higher mean score for LUTS than normal-weight children. The prevalence of overactive bladder increased as waist or BMI increased, although the relationship varies by gender (77).

### Lower Urinary Tract Symptoms in Metabolic Syndrome

Metabolic syndrome (MetS) is a cluster of metabolic disorders that include central obesity, dyslipidemia, insulin resistance, and arterial hypertension. The prevalence of MetS in the United States increased from 32.9% in 2003–2004 to 34.7% in 2011–2012 (78). The association between LUTS and MetS is well established in many clinical and experimental studies. Patients with MetS have higher risks of LUTS, including incomplete emptying, intermittency, and nocturia (79). Multiple studies have reported associations between metabolic factors and higher American Urological Association Symptom Index scores in men (18, 69, 79, 80). In the Boston Area Community Health Survey, Kupelian et al. analyzed data on 1,899 men between the ages of 30–79 and found men with mild to severe LUTS had higher odds of MetS (79). The prevalence of MetS was increased from 20% with no symptoms or one symptom to about 40% with mild to severe LUTS (79). In another study, Zamuner et al. found a 2-fold increased risk for LUTS in male patients with MetS (81). MetS may predispose patients to BPH. A meta-analysis of eight studies, which enrolled 5,403 patients, suggests that BPH is highly linked to MetS and its individual component (82). Old and obese patients have a higher risk of developing an enlarged prostate (82). Similar findings were obtained from another meta-analysis including 52 studies, in which the authors demonstrated that MetS-induced inflammation is a potential contributor to BPH (83).

### Lower Urinary Tract Symptoms in Diabetes

The CDC estimated that 34.2 million people, or 10.5% of the population in the United States, had diabetes in 2018, and approximately 90–95% of cases have type 2 diabetes. Multiple studies have shown a strong association between LUTS and diabetes. It has been estimated that between 25 and 87% of patients with diabetes experience some types of bladder dysfunction, including LUTS (84). The different etiology and pathophysiology of the two types of diabetes may give rise to different phenotypes of LUTS. In Diabetes Control and Complications Trial/Epidemiology of Diabetes Interventions and Complications (DCCT/EDIC) study including 652 women and 713 men with type 1 diabetes, the prevalence of LUTS is different in women (22%) and men (25%) (85). The risk of LUTS is increased in men with diabetes by 25% to threefold (86). Van Den Eeden et al. (87) analyzed the combined data from the California Men’s Health Study (CMHS) including 78,273 men, and the Research Program and Genes, Environment and Health (RPGEH) study including 106,373 men. They found that type 2 diabetes is associated with a 1.32-fold increased risk of LUTS in men (87). Another study has reported that men with type 2 diabetes had high storage and postmicturition symptom scores than age- and prostate volume-matched controls (88). In a survey of 2,115 white men aged 40–79 years, Burke et al. found the American Urological Association Symptom Index changed annually in patients with diabetes, indicating diabetes was associated with LUTS (89). It has been shown that women with type 2 diabetes are more likely to experience urinary incontinence compared with control subjects, although these studies fail to differentiate the different kinds of urinary incontinence (urge, stress, mixed, or overflow) (28, 90). Moreover, the prevalence of OAB in diabetic women is much higher than that of healthy controls (91, 92). LUTS in patients with diabetes are critically impacted by gender, possibly due to hormone variations and their effects on LUT organs, as well as the differences in physiology and anatomy between the male and female urethra (93).

### Lower Urinary Tract Symptoms in Cardiovascular Diseases

Cardiovascular diseases (CVDs) refer to disorders of the heart or blood vessels and mainly include coronary heart disease, stroke, aneurysms, and other heart- and blood vessel-related diseases. CVDs are the leading cause of death worldwide. World Health Organization (WHO) estimated 17.9 million people died from CVDs globally in 2016, accounting for 31% of all deaths, and most cases (85%) died from a heart attack or stroke. There are many lines of evidence supporting the association between LUTS and cardiovascular events (94, 95). Kupelian et al. (32) reported a correlation between heart disease and the duration and severity of LUTS represented by nocturia and urinary frequency in men, while weak stream and strain in women. The correlation between urologic symptoms and heart disease has been shown to vary by gender, presumably due to the different responses of the male and female urinary tracts to the pathological condition (32). Whether LUTS severity can be a significant risk factor of cardiovascular disease has been investigated in some studies (35), and the findings were controversial. The meta-analysis results from pooled 15 studies revealed that moderate to severe LUTS could indicate the increased risk of cardiac disease in the male population (34). Obese men and women with LUTS are more likely to experience heart disease (33). On the contrary, another meta-analysis performed on five longitudinal studies does not confirm that LUTS can predict cardiac events in men without cardiovascular disease history (96).

Lower urinary tract symptoms are more common among stroke patients and have a significant impact on the quality of life. Nocturia (76%), urgency (70%), and daytime frequency (59%) are the most frequent symptoms in stroke patients (97). Tian et al. have shown that LUTS prevalence in patients with stroke history is about 62.6% (15). Storage symptoms in stroke patients have been found to be significantly correlated with increasing age, male gender, high education, living alone, snoring, and co-existing medical conditions such as hypertension, and coronary heart disease (15). While, voiding symptoms, including slow stream and straining, are markedly associated with age and physical activity following stroke (98).

### Lower Urinary Tract Symptoms in Hypertension

The National Health and Nutrition Examination Survey in 2017–2018 revealed that the prevalence of hypertension was 51.0% in men and 39.7% in women. The prevalence of hypertension increased with age, from 22.4% of patients aged 18–39, 54.5% of patients aged 40–59, to 74.5% among those aged 60 and over (99).

Emerging evidence highlights hypertension as one of the risk factors that increase the frequency and severity of LUTS (100). Hypertension was highly associated with nocturnal polyuria in both genders (101, 102). A study by Hwang et al. has demonstrated that hypertensive patients have a higher International Prostate Symptom Score (IPSS) and larger prostate volume than normotensive men, indicating the association between hypertension and male LUTS (37). Chong et al. found hypertension was significantly associated with moderate-to-severe LUTS after adjusting for demographic and lifestyle factors (103). In a multicenter study in Japan including 10,744 men with LUTS, hypertension was the most common comorbidity (25.9%). The prevalence of hypertension was related to the degree of frequency and nocturia (38). Moreover, hypertension has been shown to worsen LUTS and may decrease the effectiveness of α1-blocker and terazosin therapy on BPH (39).

### Lower Urinary Tract Symptoms in Obstructive Sleep Apnea

Obstructive Sleep Apnea (OSA) is a common respiratory disorder characterized by episodes of partial or complete obstruction of the upper airway during sleep. It was estimated that OSA may affect approximately 1 billion of the world’s adult population (7.3 billion) and is even continuing to rising (104, 105). Emerging evidence suggests a possible link between LUTS and OSA. The prevalence of nocturia in patients with OSA was from 52 to 70% depending on OSA severity (40–45), and the frequency of nocturia is directly proportional to the severity of OSA (46). The results from the Complementary and Alternative Medicine for Urological Symptoms (CAMUS) trial revealed that the severity of obstructive/voiding LUTS in men was significantly associated with sleep disturbance (106). Continuous Positive Airway Pressure therapy (CPAP) is an effective and widely used method for treating OSA. CPAP treatment can decrease the frequency of nocturia and therefore improve the quality of life (45, 107).

### Lower Urinary Tract Symptoms in Autoimmune Diseases

Autoimmune diseases arise when the immune system cannot differentiate between self and foreign antigens, and both genetic and environmental factors are involved. More than 80 different autoimmune diseases affect nearly 4% of the world’s population. The prevalence in the United States is around 7%, or up to 23.5 million. Common autoimmune diseases include Sjögren’s syndrome (SS), systemic lupus erythematosus (SLE), and multiple sclerosis (MS).

The prevalence of severe LUTS is higher in patients with autoimmune diseases than in healthy people. Haarala et al. (47) studied the prevalence of urinary symptoms in patients with SS (*n* = 36), SLE (*n* = 85), and 121 control subjects. Among the three groups, 61, 62, and 27% had mild symptoms, and 14, 9, and 7% had severe symptoms. Urinary frequency (27 and 62%, respectively) and suprapubic pain (36 and 34%, respectively) were mostly reported in SS and SLE patients (47). In addition, patients with SLE and SS have significantly worse overactive bladder symptoms compared to controls (50). LUTS are also commonly reported among MS patients and usually appear 6–8 years after the initial diagnosis. After 10 years of duration, more than 90% of MS patients report LUTS, and the symptom severity usually correlates with the disability status of patients (48, 49).

### Lower Urinary Tract Symptoms in Alzheimer’s Disease

Alzheimer’s Disease (AD) is the most prevalent neurodegenerative disease accounting for dementia among older adults. It is characterized by the deposition of tau, neurofibrillary tangles, and beta-amyloid plaques in the brain, clinically showing a progressive loss of memory and higher executive functioning (108). Data from Alzheimer’s Association estimated that 5.8 million Americans are suffering from Alzheimer’s dementia in 2019. The prevalence of Alzheimer’s dementia increased with age: from 3% among adults aged 65–74, to 17% among aged 75–84, and 32% among those aged 85 and older (109).

The association between AD and LUTS was reported in several clinical studies. Takahashi et al. found AD was an independent risk factor of OAB and urinary incontinence in older people (51). The risk of urinary incontinence (UI) is higher in AD cohort (hazard ratio: 1.54, 95% confidence interval: 1.13–2.09) than in the control group (110). A clinical study conducted by Na et al. revealed that about 25% of patients with AD experienced UI (52). Recently, Jung et al. (53) have demonstrated that OAB is more common in patients with AD than control subjects, and the prevalence of OAB symptoms increases with age. In addition, the severity of OAB symptoms has been shown to be correlated with the severity of AD (53).

### Lower Urinary Tract Symptoms in Parkinson’s Disease

Parkinson’s disease (PD) is the second most common degenerative neurological disease after AD, characterized by the progressive loss of dopamine (DA) neurons in the substantia nigra pars compacta, ultimately leading to debilitating symptoms like resting tremor, muscular rigidity, and postural imbalance. Parkinson’s Foundation estimated that 7–10 million people globally have PD, and nearly 1 million Americans suffer from PD.

Lower urinary tract symptoms are frequently observed in PD, affecting 27–85% of PD patients and significantly impacting their quality of life (111–113). Benli et al. reported significantly higher urologic complaints in PD patients than in age-matched controls (114). Patients with PD have storage problems or voiding problems, or both (54). Yeo et al. reported about 57–83% of PD patients had storage symptoms, whereas 17–27% of patients experienced voiding symptoms (115).

## Systemic Oxidative Stress is Implicated in Aging and Major Chronic Diseases

### Oxidative Stress in Aging

Aging is a process characterized by a progressive functional decline of all body organs. Among the proposed aging theories, the most plausible one is the free radical theory or the oxidative stress theory. In a study of 100 healthy men and women in five age groups, Kasapoglu et al. found protein carbonyl content increased in subjects aged 60–69 years compared to those aged 20–29 and 30–39 years old (116). The activities of the main antioxidative enzymes in blood including Cu, Zn-SOD, GPx, catalase decreased significantly in older people (65–90+ years) compared to the younger group (aged 55–59 years) (117). Another study showed that the activation response of Nrf2 signaling to OS and the expression of its target antioxidant genes decreased in an age-dependent manner (118). The decrease of mitochondrial oxidative phosphorylation in aging may contribute to the increase in the production of ROS. The precise mechanisms of OS-induced aging are still not completely understood. The progressive aging-related functional loss may be associated with accumulated oxidative damage of all types of biological molecules, including lipids, DNA, and protein (119). Increased free radicals may cause cellular senescence, therefore preventing cellular proliferation processes in response to injury. In addition, senescent cells gain an irreversible senescence-associated secretory phenotype (SASP), secreting soluble factors (such as chemokines and interleukins) and matrix-degrading enzymes, altering the microenvironment and affecting the behavior of the surrounding cells (120, 121).

### Oxidative Stress in Obesity

Both experimental and clinical studies have shown that obesity and OS are strongly interconnected, despite being difficult to define the temporal sequence of their association (122). Gutierrez-Lopez et al. (123) found plasma OS markers including malondialdehyde (MDA), carbonyl group, and dityrosine increased in obese patients (BMI, 30–34.9 kg/m^2^) compared to control subjects (BMI, 18.5–24.9 kg/m^2^). These changes could be reversed by treatment with a hypocaloric diet for 90 days (123). Several mechanisms have recently been proposed to explain the augmented OS during obesity, including altered glucose and lipid homeostasis, systemic inflammation, hyperleptinemia, and abnormal postprandial ROS production (122). On the other hand, the systemic OS can induce obesity by stimulating pre-adipocyte proliferation and adipocyte differentiation and increasing the size of mature adipocytes (124). Furthermore, OS has been postulated to be an important mechanism underlying obesity-related MetS (125).

### Oxidative Stress in Metabolic Syndrome

Metabolic syndrome is strongly associated with chronic systemic OS (126). Van Guilder et al. examined the plasma biomarkers of OS in 48 normal-weight, 20 obese, and 20 obese with MetS adults (127). They found that the plasma concentrations of oxidized low-density lipoprotein inhere groups are 45.1 ± 1.8, 54.0 ± 4.0, and 62.3 ± 3.2 U/L, respectively, indicating increased OS levels in obesity and further in MetS. In another study, Jialal et al. found increased levels of oxidized low-density lipoprotein (Ox-LDL) and nitrotyrosine in plasma, and enhanced superoxide anion release from monocytes in MetS patients compared with controls (128).

Increased ROS production and decreased potency of the free radical scavenging system contribute to the increased OS level in MetS. High energy diet increases the mitochondria’s metabolic load, resulting in an overactive electron transport chain that generates excessive ROS. The activity of NADPH oxidase (Nicotinamide adenine dinucleotide phosphate oxidase, NOX) is upregulated in the aorta, kidney, and adipose tissues in high fat diet-fed rats, leading to excessive production of superoxide ions – a major oxygen free radical (129). On the other hand, MetS-induced OS may be related to impaired antioxidative activity (130). Lucie et al. examined the activities of antioxidant enzymes in hemolysed erythrocytes. They found increased activities of Cu, Zn-SOD, and glutathione reductase, while decreased activities of catalase and paraoxonase 1 in the MetS group compared to healthy controls, indicating a disorder in antioxidant defense mechanism (131).

### Oxidative Stress in Diabetes

The redox imbalance between the production of ROS and antioxidant defense systems is also associated with the development of diabetes and its complications. Metabolic abnormalities in diabetes cause excessive production of ROS and RNS, which leads to OS and cellular damage (132, 133). MDA, protein carbonyl (134), and 8-hydroxy-2′-deoxyguanosine (8-OHdG) (135) increased consistently in patients with type 2 diabetes compared to healthy subjects. In addition, reduced total antioxidant status was also observed in type 2 diabetic patients compared to controls and prediabetic patients (136). Several sources of ROS have been identified during diabetes, including enhanced blood glucose flux through the polyol and hexosamine pathway, increased formation of advanced glycation end products, and activation of protein kinase C pathway (137, 138).

### Oxidative Stress in Cardiovascular Diseases

Oxidative stress is one of the important mechanisms for the development and progression of CVDs (139, 140). The major contributors of ROS production during CVDs are mitochondrial NOX (141), lypoxidases (142), xanthine oxidase (XO) (143), and myeloperoxidases (144). In a study including 50 coronary heart disease (CHD) patients and 50 healthy volunteers, Mostafa et al. found serum GSH level, and GPx and catalase activities were significantly lower, whereas MDA level was higher in CHD patients than in healthy individuals (145). Patel et al. (146) measured the plasma levels of oxidized and reduced cystine and GSH in 1,411 patients undergoing coronary angiography. They found a high extracellular oxidant burden or reduced intracellular antioxidant capacity is associated with higher mortality in patients with coronary artery disease (146).

Oxidative stress has emerged as a key detrimental player in ischemic stroke. Impaired antioxidant defense system has also been observed in ischemic stroke patients (147). During brain ischemia, oxygen depletion stimulates the glucolytic pathway as an anaerobic source of ATP production, resulting in lactic acid accumulation and acidosis. The latter promotes pro-oxidant production and oxidative injury to neurons (148). Milanlioglu et al. reported higher levels of serum MDA and lower levels of antioxidant enzyme activity in acute ischemic stroke patients than in healthy subjects (149). Laura et al. measured the OS markers in 50 patients with acute ischemic stroke (150). They found the levels of lipoperoxide, hydroperoxide, and conjugated diene in platelets were significantly higher in the early stage and positively correlated with the NIH Stroke Scale/Score (NIHSS).

### Oxidative Stress in Hypertension

Oxidative stress plays an important role in the pathogenesis of hypertension. Lip et al. have shown that patients with malignant hypertension exhibited increased OS damage, manifested by higher levels of serum lipid hydroproxides, compared with normotensive subjects (151). Furthermore, the activities of endogenous antioxidant enzymes including SOD and GPx in erythrocytes and whole blood were markedly suppressed in untreated mild hypertensive patients than in age-matched healthy controls (152). Vascular ROS can be produced in both endothelium and smooth muscle. The major enzymatic systems involved in ROS production during hypertension include NOX, XO, endothelial nitric oxide synthase (eNOS) uncoupling, cytochrome P450 epoxygenase, and cyclooxygenase (153). Non-phagocytic NOX is potentially activated by angiotensin II during hypertension, causing the excessive production of hydrogen peroxide, superoxide, and peroxynitrite (154). eNOS uncoupling is caused by deficiency or oxidation of tetrahydrobiopterin, an important cofactor for eNOS action, leading to increased generation of superoxide and decreased NO bioavailability (155). XO has been shown to underlie the development of hypertension-induced end-organ damage (156). In addition, ROS generated in mitochondria and the endoplasmic reticulum also contribute to the development of hypertension (157, 158).

### Oxidative Stress in Obstructive Sleep Apnea

A number of studies have shown that OS is present in patients with OSA. The circulating polymorphonuclear neutrophils from patients with OSA released more superoxide radical anions, possibly due to the increased activation of NOX (159). 8-isoprostane increased significantly in exhaled breath condensate of OSA patients (160). Serkan et al. (161) measured plasma total antioxidative capacity and total oxidative status in 25 healthy volunteers and 59 obstructive sleep apnea patients. They found the total antioxidative capacity was significantly lower, but the total oxidative status was significantly higher in OSA patients than the healthy controls (161). Ekin et al. found the serum levels of NOX4, ischemia-modified albumin (IMA), MDA, and 8-OHdG in OSA patients were significantly higher than those in healthy subjects (162), which might be due to cyclic hypoxia and reoxygenation in OSA. The salivary levels of OS markers, including thiobarbituric acid reacting substances, advanced oxidation protein products, and advanced glycation end products (AGEs), in patients with severe OSA were higher in the morning compared to the past evening. The change can be partially reversed by short-term continuous positive airway pressure (CPAP) (163).

### Oxidative Stress in Autoimmune Diseases

Excessive OS and/or defective antioxidant potential were also reported in patients with autoimmune diseases. In a cross-sectional study of 15 healthy subjects and 26 SS patients, Norheim et al. found plasma levels of protein carbonyl and advanced oxidation protein products were higher in the SS patients than in healthy subjects, indicating increased levels of OS (164). A strong association between the development of SLE and the polymorphisms of genes encoding NADPH and several antioxidant enzymes including SOD, GPx, and catalase, has been reported (165). In a study of nine controls and 21 SLE patients for up to 38 months, Morgan et al. found the serum levels of protein carbonyls were correlated with disease activity (166). Mitochondrial dysfunction, which can cause an overproduction of ROS and RNS, was observed in the early stage of MS patients (167). The urinary levels of 8-iso-PGF2α (a marker of lipid peroxidation) were higher in MS patients than in healthy controls (168). OS is involved in MS through both central and peripheral mechanisms (169). ROS-induced damage contributes to the demyelination in MS. At the same time, ROS-mediated pathways regulate immune cell priming in peripheral lymphoid organs. T cells traveled to the brain parenchyma can be activated by microglia or macrophages, promoting inflammatory cascade (169).

### Oxidative Stress in Alzheimer’s Disease

The brain requires an abundant supply of both oxygen and glucose since the oxidation of glucose under aerobic conditions is the primary energy source for the brain. On the other hand, the brain is more vulnerable to OS because it has abundant, easily oxidizable lipid (170). Previous studies found reduced glucose metabolism and impaired oxygen consumption in the brains of AD patients, indicating mitochondrial dysfunction (171). Mitochondrial dysfunction and associated accumulation of ROS may participate in AD development. The levels of OS markers, including protein carbonyls, 4-hydroxy-2-non-enal (HNE), 3-nitrotyrosine (3-NT), and 8-OHdG, are increased in the blood and cerebrospinal fluid of patients with AD (172, 173). OS can cause neuron injury and degeneration partially through the release of excitatory amino acids and an increase in intracellular free Ca^2+^ (174). Therefore, OS has been considered one of the causative factors for developing AD (175).

### Oxidative Stress in Parkinson’s Disease

Oxidative stress has been demonstrated to play a role in dopaminergic cell death in PD. Markers of lipid peroxidation, nucleic acid, and protein oxidation, along with reduced levels of GSH have been found in dopaminergic neurons of PD patient (176). A recent meta-analysis, including 7,212 PD patients and 6,037 healthy subjects from 80 studies, found the levels of blood MDA and 8-OhdG were increased, but catalase and GSH were decreased in PD patients compared with controls (177). The primary sources of ROS in PD include dopamine neurotransmitter metabolism, endoplasmic reticulum stress, mitochondrial dysfunction, and neuroinflammation (178). The nigral dopaminergic neurons contain iron which catalyzes the Fenton reaction where ferrous iron (Fe^2+^) reduces H_2_O_2_ to yield hydroxyl radical (HO⋅), a highly reactive ROS. Besides, dopaminergic neurons are susceptible to OS and have ROS-producing enzymes, such as, tyrosine hydroxylase and monoamine oxidase. For these reasons, even moderate OS can trigger a series of events that ultimately lead to cellular death (179).

## Impact of Oxidative Stress on the Lower Urinary Tract

### Impact of Oxidative Stress on the Urinary Bladder

Several lines of evidence support that OS is a major pathogenic factor underlying the development and progression of bladder dysfunction in certain pathologic conditions, including diabetic cystopathy (180), bladder outlet obstruction (181), and chronic bladder ischemia (182). ROS can damage detrusor muscle mitochondria, resulting in decreased efficacy of energy production and impaired bladder contraction (183).

In previous studies, we found increased nitrotyrosine levels in the bladders of 9-week diabetic mice (184), and 20-week (185) and 44-week (186) diabetic rats. To investigate the role of OS on bladder dysfunction, we generated a mouse model with inducible smooth muscle (SM) - specific deletion of manganese superoxide dismutase (MnSOD), encoded by the superoxide dismutase-2 gene (*Sod2*). The depletion of MnSOD caused increased nitrotyrosine expression in the bladder, indicating OS. Meanwhile, the SM-specific *Sod2* knockout mice had overactive bladder symptoms (187). One possible explanation for OS-induced bladder hyperactivity may be related to the activation of C fiber afferent pathway (188). OS has also been shown to mediate bladder hyperactivity in a chronic bladder ischemia rat model induced by atherosclerosis (189–191), possibly through affecting the release of nerve growth factor and the expression of its receptor p75, leading to neurodegeneration and neuropathy in the bladder (192). On the other hand, the severe and prolonged OS has been shown to impair detrusor contractility, leading to bladder underactivity (193, 194). Therefore, it is conceivable that the effects of ROS/RNS on bladder function are dose and/or duration-dependent. Induction of mild OS in the bladder of rats by intravesical administration of a low dose of H_2_O_2_ (0.003%) has been found to cause higher peak voiding pressure and detrusor hyperactivity. In contrast, a high dose of H_2_O_2_ (3%) induced a decline of the peak voiding pressure and impaired detrusor contractility (188). The mild OS may activate the bladder afferent nerves (188) and stimulate the detrusor muscle cells directly (195), leading to bladder hyperactivity, while severe or prolonged OS can induce detrimental alterations in nerves and the detrusor muscle ultimately resulting in detrusor underactivity (196).

### Impact of Oxidative Stress on the Urethra

Oxidative stress has emerged as a potential pathogenic factor for urethral dysfunction in diabetes (197, 198) and aging (199). Urethral dysfunction can manifest as the impairment of contraction or relaxation (200, 201). The former is related to urinary incontinence, while the latter leads to urethral tightening and functional outlet obstruction, lowering the voiding efficiency. OS significantly attenuates the generation and bioavailability of NO, a major transmitter that induces urethral relaxation during bladder voiding (202). In addition, overproduction of ROS has been shown to induce oxidative injury and cause atrophy of the urethral sphincter muscles, leading to impaired urethral closure (198). Enhanced OS coupled with increased collagen deposition has been observed in rats with castration, and the changes can be attenuated by alpha-tocopherol supplementation (203). Alexandre et al. (204) have reported higher levels of ROS, along with decreased levels of NO and SOD in the urethral smooth muscle of obese mice. All these abnormalities are reversed by treatment with resveratrol (204). In a streptozotocin-induced diabetic rat model, urethral dysfunction was associated with increased OS markers, decreased activity of antioxidant enzymes, along with the downregulation of nNOS, NO, and cGMP (197).

### Impact of Oxidative Stress on the Prostate

There is increasing evidence highlighting OS as an important mechanism that triggers a series of events involved in the development and progression of BPH. Patients with BPH have higher plasma and urine levels of OS markers than age-matched healthy subjects (205, 206). Resveratrol is a nutraceutical belonging to the polyphenols’ stilbenoids group, mostly found in grapes’ skin and seeds, some berries, and peanuts. Numerous studies have demonstrated that resveratrol possesses anti-inflammatory and antioxidant properties (207). After 2-months of treatment with resveratrol, patients with prostate fibrosis and LUTS showed significant improvement of NIH-Chronic Prostatic Symptom Index (NIH-CPSI) and IPSS scores (208). In another clinical study, Matsumoto et al. (209) used Eviprostat, a phytotherapeutic agent composed of several plant extracts, to treat BPH-associated LUTS. They found the total IPSS decreased from 16.56 ± 2.74 to 13.67 ± 2.30 after Eviprostat treatment. This effect is accompanied by a 2.5-fold decrease of urinary 8-OHdG. The authors concluded that the antioxidant activity of Eviprostat may be responsible for its treatment effects (209).

The causal role of OS in BPH has been supported by Vital et al.’s (210) study, in which they generated a transgenic mouse model with overexpression of Nox4 under the control of the prostate-specific promoter ARR2PB. The transgenic ARR2PB-Nox4 mice were found to display oxidative injury in prostate tissue, prostate hyperplasia along with histological remodeling (epithelial proliferation and fibrosis) (210). Several plausible mechanisms have been proposed to understand the role of OS in causing BPH. OS induces oxidative DNA damage, adversely affecting the balance between cell proliferation and programmed cell death leading to hyperplasia. In addition, NF-κB is the master inflammatory transcriptional regulator of genes involved in inflammation, cell proliferation, cell migration, and apoptosis (211). ROS can activate the NF-κB pathway, contributing to chronic prostate inflammation and BPH (212). Furthermore, OS is associated with increased plasma activity of prolidase, a key enzyme in collagen turnover and matrix remodeling, promoting the development of BPH (213).

## Conclusion

**FIGURE 1:**
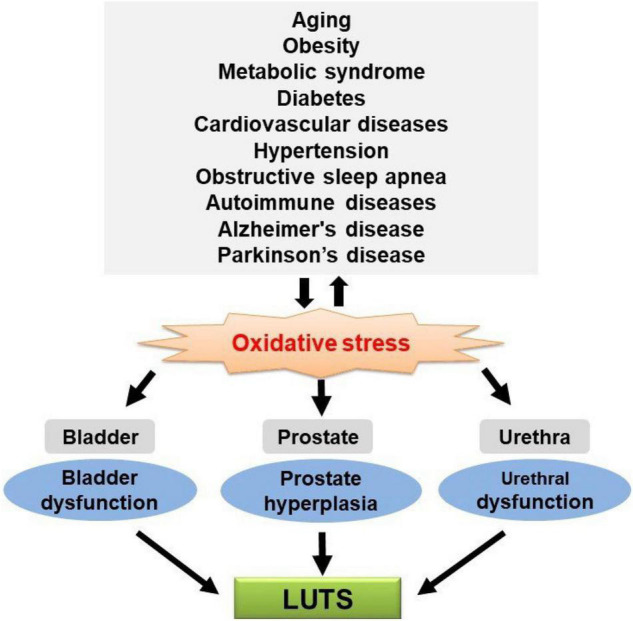
Oxidative stress (OS) is a putative mechanistic link between lower urinary tract symptoms (LUTS) and aging and chronic diseases.

Many studies showed that LUTS are highly prevalent in the aging population and patients with chronic diseases, supporting the speculation that those disease-related pathophysiological changes also contribute to the development of LUTS. There may be one or more common underlying factors for the aforementioned chronic diseases and LUTS. Studies also showed that OS is a mutual pathogenic factor of aging and major chronic diseases. Meanwhile, recent studies showed that exacerbated OS could cause functional and/or structural changes in the bladder, urethra, and prostate, leading to LUTS. Taken together, these data suggest that OS seems to be a potential link between LUTS and aging and major chronic diseases (Figure 1), although further studies are needed to confirm the causative relationship. Urologists should consider factors outside the lower urinary tract that may be contributing to LUTS and the higher likelihood of LUTS during aging and these major chronic diseases. It remains to be determined whether treatment of these diseases can significantly improve urologic symptoms. On the other hand, general antioxidant treatment might not be the best choice since ROS/RNS also serve as second messengers in the physiological regulation of intracellular signaling cascades involved in various cellular functions. Future investigation is required to identify the specific ROS/RNS and corresponding reactions/pathways involved in aging and chronic diseases and associated LUTS, which would be a reasonable basis for developing particular antioxidants for clinical application.

## Author Contributions

GL: conceptualization and writing—review and editing. ZX, RE, BL, and GL: writing—original draft preparation, read, and agreed to the published version of the manuscript.

## Conflict of Interest

The authors declare that the research was conducted in the absence of any commercial or financial relationships that could be construed as a potential conflict of interest.

## Publisher’s Note

All claims expressed in this article are solely those of the authors and do not necessarily represent those of their affiliated organizations, or those of the publisher, the editors and the reviewers. Any product that may be evaluated in this article, or claim that may be made by its manufacturer, is not guaranteed or endorsed by the publisher.
